# Dynamic and Steady Characteristics of Polymer-Ceramic Pressure-Sensitive Paint with Variation in Layer Thickness

**DOI:** 10.3390/s17051125

**Published:** 2017-05-15

**Authors:** Tatsunori Hayashi, Hirotaka Sakaue

**Affiliations:** Hessert Laboratory for Aerospace Research, Department of Aerospace and Mechanical Engineering, University of Notre Dame, Notre Dame, IN 46556, USA; thayashi@nd.edu

**Keywords:** unsteady pressure-sensitive paint, thickness, fast response

## Abstract

Polymer-ceramic pressure-sensitive paint (PC-PSP) has been investigated as a surface-pressure sensor for unsteady aerodynamics and short duration measurements. This PSP provides a fast response to a change in pressures with a spray-coating ability. Because it is sprayed onto an aerodynamic surface, the thickness of PC-PSP may play an important role in determining the performance of this sensor. The thickness of other fast PSPs, such as anodized aluminum pressure-sensitive paint, is a major factor in determining its performance. We vary the thickness of PC-PSP from 10 to 240 μm in order to study its effects on PSP measurement characteristics including time response, signal level, pressure sensitivity, and temperature dependency. It is found that the thickness does affect these characteristics. However, a thickness over 80 μm provides uniform performance in these characteristics.

## 1. Introduction

**Figure 1:**
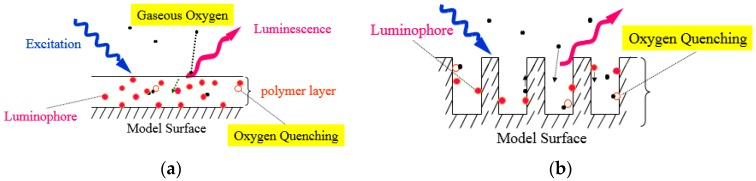
Schematic description of (**a**) a polymer pressure-sensitive paint (PSP) and (**b**) a porous PSP.

Pressure-sensitive paint (PSP) has been widely utilized in aerospace applications [1,2,3,4,5,6,7,8,9,10]. In combination with a fast frame-rate camera, PSP measurement opens its application to unsteady flow measurement on surfaces and to short duration testing [11,12,13,14,15]. PSP uses a photophysical process of oxygen quenching to relate an oxygen pressure of a testing fluid to a luminescent signal. A luminophore and a supporting matrix that is mixed solvent with a polymer and chemical solvent configure the PSP. The former gives a luminescent signal and the latter holds the luminophore onto a test piece. Oxygen quenching is key for relating the luminescent output to the pressure [16]. The supporting matrix greatly affects the response time of a PSP [17,18]. A conventional PSP uses a polymer as a supporting matrix, which can be sprayed onto any testing model (Figure 1a). To cause oxygen quenching, gaseous oxygen needs to permeate into this layer. Due to the polymer aero-permeability, this type of PSP shows a time response of the order of seconds to sub-seconds. Using a porous material as a supporting matrix enhances the response, as pores or other structures are open to a test gas (Figure 1b). This porous structure provides PSP with a fast response to a pressure change by enhancing mass diffusion in pores. This porous surface structure results in an enhanced time response of this PSP of approximately ten microseconds [18,19,20]. However, this porous PSP is constrained by the supporting matrix [21]. Anodized-aluminum pressure-sensitive paint (AA-PSP), for instance, can only be applied to aluminum models or test pieces [21]. There is a need to combine fast response characteristics with the ability to spray the paint onto any test article. 

**Figure 2:**
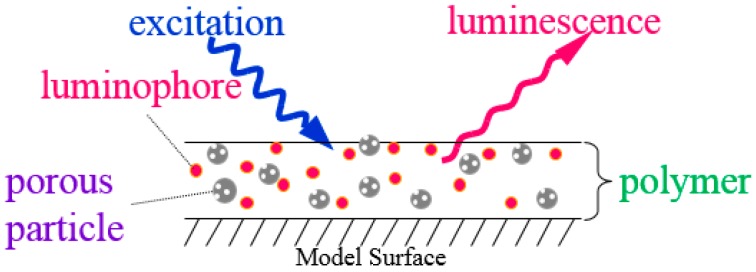
Schematic description of a polymer-ceramic PSP (PC-PSP).

Polymer-ceramic PSP (PC-PSP) has been investigated to satisfy both the fast response and spraying characteristics [22,23,24,25,26]. This PSP uses both a porous material and polymer as a supporting matrix (Figure 2). The former enhances the time response, and the latter provides the spraying ability.

There are some studies discussing the time response related to the PSP thickness [5,6,23,27]. In general, the time response is proportional to the squared value of the thickness [27]. Because PC-PSP is a combination of porous particles and a polymer, the trend in the time response would not follow the previously reported results. It is necessary to understand the time response of a PC-PSP to use this tool to capture the flow fields in unsteady aerodynamic measurements and short duration testing. Based on the above-mentioned literature, the PSP thickness will be a major factor influencing the response time of a PC-PSP. In this paper, we focused on the influence of the thickness of PC-PSP. To study the influence of the thickness, it was varied from 10 to 240 μm.

## 2. Experimental Setups and Methods

### 2.1. Materials

PC-PSP consists of a luminophore, porous particle, polymer, and solvent. Silica gel (SiO_2_, Sigma-Aldrich, St. Louis, MO, USA) was chosen as a porous particle. PC-PSP was prepared based on the method described by Sakaue et al. [22]. The porous particles had a mean particle size between 2 and 25 μm. Room Temperature Vulcanizing (RTV) rubber (KE-41, Shin-Etsu Chemical, Tokyo, Japan) was used as a polymer. The dichloromethane that was the organic solvent decomposed the aggregation of the particles and dissolved the polymer to mix these components. Then, the ultrasonicator was performed for 20 min to reduce the porous particle aggregation of the polymer-particle mixed solvent, and the ultrasonicated solvent was sprayed over the 20-mm square aluminum test plate. After spraying, the mixing solvent was evaporated. To study the influence of changing the coating thickness on PC-PSP characteristics, we adjusted the thickness of the polymer-ceramic coating from 10 to 240 μm, which was measured by an eddy current apparatus (LZ-300, Kett Electric Laboratory, Tokyo, Japan), and thickness was adjusted between ±3 μm. These PC-PSPs were named *PCPSP_th_*_01_, *PCPSP_th_*_03_, *PCPSP_th_*_08_, *PCPSP_th_*_13_ and *PCPSP_th_*_24_, relevant to their thickness. The polymer to particle ratio (polymer content) was set at 20 wt %. Bathophen ruthenium from GFS Chemicals was used as a luminophore. It was dissolved in acetone to create 0.1 mM solution. The resultant polymer-ceramic coatings were dipped into the solution for 20 min under room conditions. The luminophore was adsorbed onto the polymer-ceramic surface, and the dipping solvent was evaporated after the dipping process. Three PC-PSP samples were prepared for each coating thickness to obtain mean and standard deviation values.

### 2.2. Steady-State Characterization

**Figure 3:**
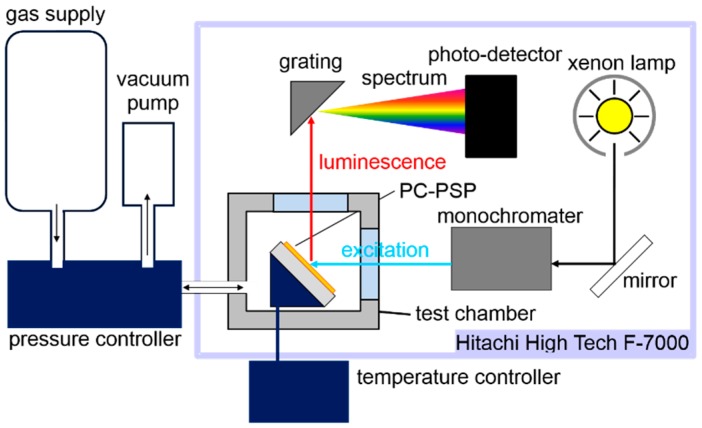
Schematic steady-state calibration setup.

The steady-state characterization system determined the signal level, pressure sensitivity, and temperature dependency of the PC-PSP based on the spectral output of a PC-PSP at a controlled pressure and temperature. As shown in Figure 3, the steady-state characterization system was composed of a spectrometer (F-7000, Hitachi High-Technologies, Tokyo, Japan) and a pressure- and temperature-controlled chamber. PSP samples were excited at 465 nm wavelength, and the excitation area covered the whole sample. The luminescent intensity of the PC-PSP was decided by the integration of the emission spectrum within 620 ± 20 nm. The reference conditions were 100 kPa and 300 K. Dry air was used to reduce the humidity influence on the PSP. All samples were put in the same optical setup during measurement to determine the signal level at the reference conditions. Based on Liu et al., the luminescent intensity, *I*, can be calculated by multiplying the gain of the photo-detector in a spectrometer, *G*, the emission from the PC-PSP, *I_PCPSP_*, the excitation in the spectrometer, *I_ex_*, and the measurement setup component, *f_set_* [28]:

(1) $$I=\;GI_{PCPSP}\;I_{ex}f_{set}$$

In our system, *G*, *I_ex_*, and *f_set_* were the same for all PC-PSP samples. We non-dimensionalized *I* by that of *PCPSP_th_*_01_. We defined the signal level, *η*, as shown in Equation (2):

(2) $$\eta =\frac{I}{I_{PCPSP_{th01}}}\left(\%\right)$$

For the pressure calibration, the pressure in the chamber, *P*, was varied from 5 to 120 kPa, with a reference temperature of 300 K. The luminescent intensity under the reference conditions, *I_ref_*, was divided by each *I* at a given pressure to derive *I_ref_/I*. This quantity can be related to pressure using the Stern-Volmer relationship [1]:

(3) $$\frac{I_{ref}}{I}=A_{P}+B_{P}\cdot P$$

Here, *A_P_* and *B_P_* are calibration constants. Due to an oxygen adsorption on the porous surface, a PSP with a porous surface would indicate a non-linear relationship [29]. Because the PC-PSP structure is a combination of porous particles and a polymer, the same model describing a porous PSP would not be physically correct. As an alternative, we used the second-order polynomial to modify Equation (3):

(4) $$\frac{I_{ref}}{I}=A_{P}+B_{P}\cdot P+C_{P}\cdot P^{2}$$

where *C_P_* is an additional calibration constant. The pressure sensitivity, *σ*, explains the change in *I* over a given pressure change. This corresponds to a slope of Equation (4) under the reference conditions:

(5) $$\sigma ={\frac{d\left(I_{ref}/I\right)}{d_{P}}|}_{P=P_{ref}}=B_{P}+2C_{P}P_{ref}\left(\%/\mathrm{kPa}\right)$$

In general, a PSP has a temperature dependency [1]. This influences *I*, which can be explained as the second-order polynomial in Equation (6):

(6) $$\frac{I}{I_{ref}}=A_{T}+B_{T}\cdot T+C_{T}\cdot T^{2}$$

*A_T_*, *B_T_*, and *C_T_* are calibration constants. To calibrate the temperature sensitivity of the samples, the temperature, *T*, was varied from 273 to 333 K with a reference pressure at 100 kPa. Equation (7) shows the slope of the temperature calibration at the reference conditions, which is defined as the temperature dependency, *δ*. A large absolute value of *δ* indicates that the intensity change with changing temperature is also large. For a pressure sensor, this characteristic is an unfavorable condition, while zero *δ* means that the PC-PSP has temperature independency, which is favorable:

(7) $${\delta =\frac{d\left(I/I_{ref}\right)}{dT}|}_{T=T_{ref}}=B_{T}+2C_{T}\cdot T_{ref}\left(\%/K\right)$$

### 2.3. Dynamic (Unsteady-State) Characterization

**Figure 4:**
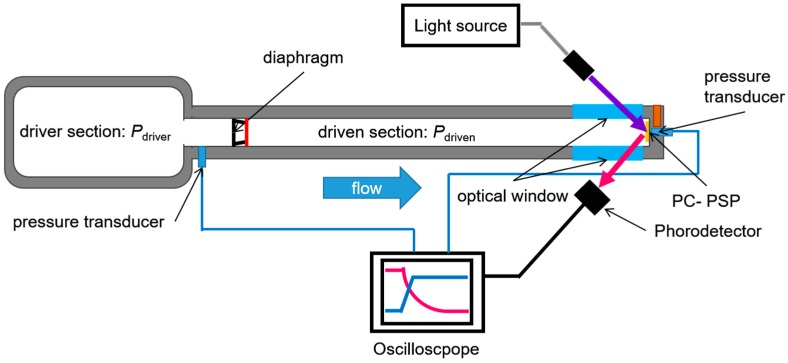
Schematic of unsteady-state calibration setup.

Figure 4 shows a schematic of the unsteady-state calibration setup, which determined the time response. The step change of pressure generated by a normal shock in a shock tube was used to determine the time response. A time delay of *I* from the step change of pressure was measured for the sensor. The driven section and driver section lengths were 1600 and 600 mm, respectively, with a cross-sectional area of 30 square mm. Dry air was used as a test gas at a reference temperature of 300 K. This shock tube had the driver and driven sections separated by a diaphragm. The diaphragm was burst by the pressure difference between the driver and driven sections, and a normal shock wave was propagated towards the PC-PSP in the driven section. The pressure difference produced by setting pressure equal to 3 kPa and 130 kPa in the driven and driver sections, respectively, produced a normal shock wave with a Mach number of 2.1 in the tube. A PC-PSP and pressure transducer (XCQ-062-50A, Kulite Semiconductor Products, Leonia, NJ, USA) were put on the end plate of the tube. An excitation light generated by a blue laser at 465 nm passed through one of the optical windows and illuminated the PC-PSP test sample. The sample emitted a luminescent intensity, *I*, and the photodetector measured it passing through the optical window on the other side of the tube. A photomultiplier tube (PMT) (H57730-04, PMT, Hamamatsu Photonics, Shizuoka, Japan) acquired its intensity, while a photodetector and a band-pass filter (620 ± 20 nm) put in front of the PMT cut off the unnecessary wavelengths. This detector was required to have a much faster time response compared to the unsteady PSP, which had a time response on the order of ten to one hundred microseconds, in order to determine the PC-PSP time response. It had a sufficient time response on the order of nanoseconds.

The Kulite sensor monitored the pressure in the driven section, *P_driven_*, and compensated for the repeatability of this characterization. After generating the shock wave, it traveled in the tube, and the PSP experienced a sharp pressure jump, *P_reflect_*, when the wave impacted the end plate. The normalized pressure, *P_norm_*, extracted the time delay, expressed as:

(8) $$P_{norm}=\frac{P-P_{driven}\;}{P_{reflect}-P_{driven}}$$

The luminescent intensity, *I*, was converted to pressure, *P*, using Equation (4).

## 3. Results and Discussion

### 3.1. Signal Level

**Figure 5:**
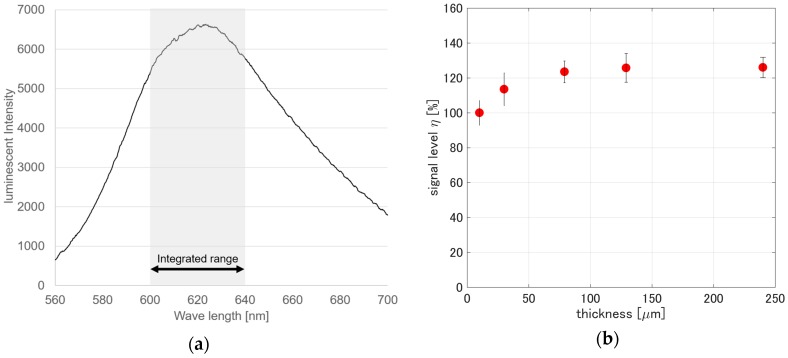
(**a**) Luminescent spectrum of *PCPSP_th_*_01_ at the reference conditions; (**b**) Signal level related to PC-PSP thickness.

Figure 5a shows a representative luminescent spectrum obtained from *PCPSP_th_*_01_ under the reference conditions, which were 100 kPa and 300 K. The integrated range shown by the shaded area determined the luminescent intensity, I, for the given pressure and temperature. The signal level, *η*, for each pressure and temperature was determined from Equation (2). The signal level, *η*, related to the thickness is shown in Figure 5b. It can be seen that as the thickness increases, *η* approaches saturation. The change in *η* for the thickness over 80 μm was within the expected level of uncertainty. With the thickness over 80 μm, we could obtain an over 20% increase of *η* compared to *η* of 10 μm in thickness. This result tells us that, if we can spray PC-PSP with a thickness over 80 μm, the intensity variation related to the thickness is minimal. This is a good aspect for a PSP measurement, because a spatial variation in luminescent intensity can be reduced by spraying PC-PSP over 80 μm. Also, the signal level saturation implied that the supporting matrix limited the amount of the luminophore per volume of the base coat layer. Even though the PSP was dipped in the solvent for a longer time than investigated here, the dipping time effectiveness on the signal level of PSP samples might not be significant.

### 3.2. Pressure Sensitivity

**Figure 6:**
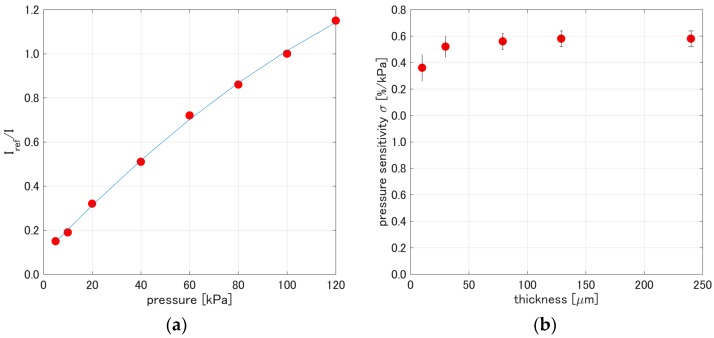
(**a**) Pressure calibration of *PCPSP_th_*_01_; (**b**) Pressure sensitivity related to PC-PSP thickness.

Figure 6a shows a representative pressure calibration obtained from *PCPSP_th_*_01_. The second-order polynomial fits well to the pressure calibration. Figure 6b shows the pressure sensitivity, *σ*, related to the thickness. The pressure sensitivity, *σ*, for each thickness was determined from Equation (5) in the pressure calibration. The *σ* ranged from 0.75 to 0.9%/kPa. Similar to the results from the signal level (Section 3.1), we see a saturation in *σ* if the thickness was over 80 μm. The change in *σ* based on *σ* of *PCPSP_th_*_01_ was over 10%. This change was smaller than that of *η*, which was over 20%. As a PSP measurement point of view, we can provide PC-PSP thickness over 30 μm to provide a uniform *σ* over the sprayed surface. As already mentioned in Section 3.1, this saturation tendency seems to agree with our suggestion that a thicker base coat layer of the PSP might produce PSP samples with stabilized characteristics. More interestingly, the pressure sensitivity can saturate a thinner PSP layer compared to the signal level.

### 3.3. Temperature Dependency

**Figure 7:**
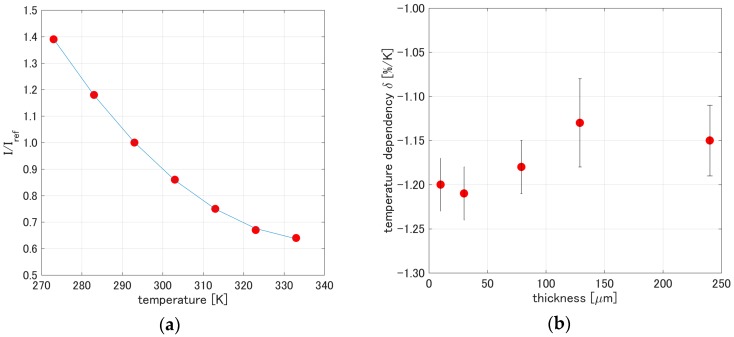
(**a**) Temperature calibration of *PCPSP_th_*_01_; (**b**) Temperature dependency related to PC-PSP thickness.

Figure 7a shows a representative temperature calibration obtained from *PCPSP_th_*_01_. A second-order polynomial could describe the temperature calibration. Figure 7b shows the temperature dependency, *δ*, related to the thickness. The temperature dependency, *δ*, for each thickness was determined by Equation (7). The *δ* decreased from −1.2%/K to −1.15%/K. The change in *δ* was within 5%, which was within the expected level of uncertainty. The results show that *δ* related to the thickness can be considered a less influential factor.

### 3.4. Time Response

**Figure 8:**
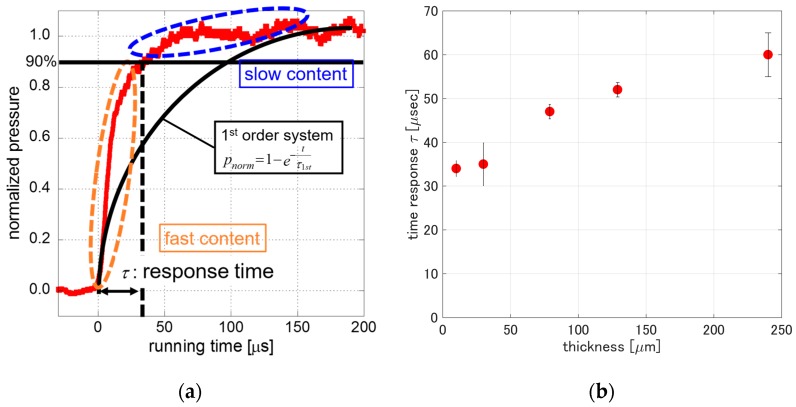
(**a**) Step response of *PCPSP_th_*_01_ following a normal shock wave; (**b**) Response time related to PC-PSP thickness.

Figure 8a shows a representative result of the step response obtained from *PCPSP_th_*_01_. The time response, *τ*, was defined as a time delay from a step change of pressure. If we use the first-order system shown in the figure, it was not appropriate to describe the response. It can be shown by fast and slow contents in the step response. Instead of applying the first-order system, we determined the time response, *τ*, as a time delay approaching the 90% change to the normalized pressure, *P_norm_* [22].

Figure 8b shows the time response, *τ*, related to the thickness. Every *τ* of varying thicknesses was calculated by the averaging of five time runs to verify the repeatability of the time response. The *τ* was monotonically increased from 35 to 60 μs as the thickness increased from 10 to 240 μm. Even though the thickness was increased by 24 times, the increase of *τ* was less than a factor of two. If we keep the PC-PSP thickness within 80 to 240 μm, the time response was within 22% change. From a measurement point of view, the present results tell us that, over 80 μm in thickness, PC-PSP provides a good uniformity in the time response as well as the signal level, pressure sensitivity, and temperature dependency.

The time response *τ* of PSP can be described by:

(9) $$\tau \propto \frac{h^{2-d_{fr}}}{D_{m}}$$

where *d_fr_* (1 ≤ *d_fr_* < 2) is the fractal dimension of the path of the pore [27]. This represents the degree of complexity of the pore path.

**Figure 9:**
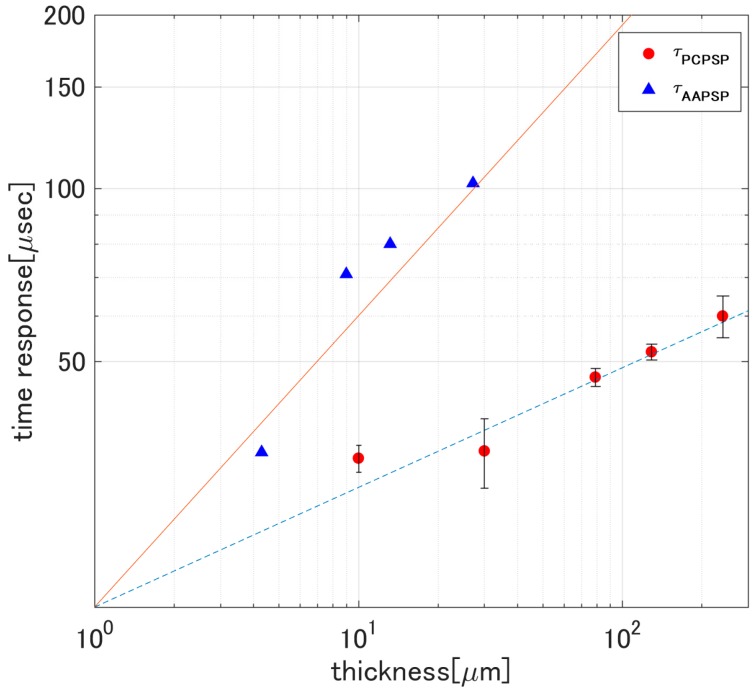
Comparison of time response for PC-PSP and AA-PSP [21].

Figure 9 overlaid the present results and the AA-PSP time response related to its thickness. The AA-PSP time response was obtained from Sakaue and Sullivan [21]. Horizontal and vertical axes are shown as a logarithmic scale to linearly fit Equation (9). Based on Equation (9), the time response of PC-PSP was proportional to *h*^0.208^, while that of AA-PSP was proportional to *h*^0.505^. Even though there was a thickness dependency on *τ*, the change in *τ* for PC-PSP was smaller than that of AA-PSP.

## 4. Conclusions

The thickness effects on polymer-ceramic pressure-sensitive paint (PC-PSP) were presented in this paper. PC-PSP has been investigated for unsteady aerodynamics and short duration testing to give surface pressure information on aerodynamic objects. It was found that the thickness changes the performance characteristics of PC-PSP, such as the signal level, pressure sensitivity, temperature dependency, and time response. These characterizations approached uniform values when the thickness was over 80 μm. From a measurement point of view, the thickness of PC-PSP can be applied greater than this thickness to yield uniform values of signal level, pressure sensitivity, temperature dependency, and time response.
